# Two Heterozygous Mutations in *NFATC1* in a Patient with Tricuspid Atresia

**DOI:** 10.1371/journal.pone.0049532

**Published:** 2012-11-30

**Authors:** Zahi Abdul-Sater, Amin Yehya, Jean Beresian, Elie Salem, Amina Kamar, Serine Baydoun, Kamel Shibbani, Ayman Soubra, Fadi Bitar, Georges Nemer

**Affiliations:** 1 Department of Biochemistry and Molecular Genetics, American University of Beirut, Beirut, Lebanon; 2 Department of Pediatrics and Adolescent Medicine, American University of Beirut, Beirut, Lebanon; University of Bonn, Institut of experimental hematology and transfusion medicine, Germany

## Abstract

Tricuspid Atresia (TA) is a rare form of congenital heart disease (CHD) with usually poor prognosis in humans. It presents as a complete absence of the right atrio-ventricular connection secured normally by the tricuspid valve. Defects in the tricuspid valve are so far not associated with any genetic locus, although mutations in numerous genes were linked to multiple forms of congenital heart disease. In the last decade, Knock-out mice have offered models for cardiologists and geneticists to study the causes of congenital disease. One such model was the *Nfatc1* ^−/−^ mice embryos which die at mid-gestation stage due to a complete absence of the valves. NFATC1 belongs to the Rel family of transcription factors members of which were shown to be implicated in gene activation, cell differentiation, and organogenesis. We have previously shown that a tandem repeat in the intronic region of *NFATC1* is associated with ventricular septal defects. In this report, we unravel for the first time a potential link between a mutation in *NFATC1* and TA. Two heterozygous missense mutations were found in the *NFATC1* gene in one indexed-case out of 19 patients with TA. The two amino-acids changes were not found neither in other patients with CHDs, nor in the control healthy population. Moreover, we showed that these mutations alter dramatically the normal function of the protein at the cellular localization, DNA binding and transcriptional levels suggesting they are disease-causing.

## Introduction

Cardiac valvulogenesis refers to the formation of valves in the heart, an evolutionary conserved mechanism in vertebrates that occurs at mid-gestation and results in the unidirectional flow of blood throughout the heart. Both the semilunar (aortic and pulmonary), and atrioventricular valves (tricuspid and mitral) are thought to arise from endocardial cells that undergo multiple processes governed by an array of growth factors, transcription factors, and extracellular proteins [1], [2], [3].

Endocardial cells destined to become valves undergo an epithelial to mesenchymal transformation (EMT) upon their stimulation by the TGFβ and BMP2/4 growth factors secreted from the underlying myocardium [2]. This process of transformation is dependent on two signaling pathways from within the endocardial cells, specifically the Wnt and NOTCH pathways [4]. The mesenchymal cells will invade the cardiac jelly composed mainly of hyaluronic acid. These cells will undergo proliferation and subsequent differentiation into mature valves, a process that is subject to tight regulation by growth factors amongst which the vascular endothelial growth factor (VEGF). The final valve structure is made up of at least 2 leaflets (mitral has 2 while tricuspid has 3) composed mainly of endocardially-derived cells. The involvement of neural crest cells in semilunar but not atrioventricular valves formation is supported by conditional knock-outs although neither myocardial nor neural crests cells are detected in the mature valves [2], [3], [5]. The final process of remodeling is governed mainly by apoptosis. Defects in any of the steps involved in valvulogenesis lead to the valvular congenital heart disease including Mitral and Tricuspid Atresia (MA and TA). These two conditions, which account for 1–2% of all congenital heart disease in humans, are still difficult to treat [6], [7].

Some of the molecular pathways involved in valve formation have been unraveled through the unexpected phenotype encountered in mice lacking the *Nfatc1* gene [8], [9]. NFATC1 (Nuclear Factor for Activated T-Cells) belongs to the Rel/NF-kB family of transcription factors that were first described as being key regulators of T-cells' activation. Five members (NFATC1-5) are found in mammals; all playing different non-redundant roles during embryonic and postnatal development [10], [11], [12], [13]. All five members share a conserved DNA-binding domain at the C-terminus of the protein that binds specifically to the consensus (A/T)GGAAA sequence [14]. In addition, they harbor at the N-terminal region a series of conserved serine-proline residues (S/P) that when dephosphorylated unmasked a nuclear localization signal allowing the translocation of NFATC proteins from the cytoplasm to the nucleus [15], [16], [17], [18]. All NFATC proteins except NFATC5 are dephosphorylated by the calcium dependent phosphatase calcineurin (PPP3CA/PPP3CB) at the N-terminus triggering the translocation process. Although NFATC proteins are weak transactivators, their transcriptional potency is boosted through their interactions with different classes of transcription factors mainly the AP-1 family members, c-Fos and Jun, the MADS family, and the GATA zinc finger proteins [19], [20], [21], [22], [23].

NFATC1 was shown to be expressed in numerous cell types including lymphocytes, osteoclasts, neurons, and myotubes [17], [24], [25], [26], [27]. The first *in vivo* assessment of the role of the gene came however from the inactivation of the gene in mice. Two independent reports showed that *Nfatc*1−/− mice die at mid-gestation stage (e14.5) due to lack of EC growth and remodeling [8], [9]. While Ranger AM et al showed a selective defect in the semilunar valves, the *Nfatc1*−/− embryos generated by de la Pompa Jl et al had severe defects in both atrioventricular and semilunar valves. Although this discrepancy might be linked to the genetic background and/or knock-out strategy, the fact that in both phenotypes the endocardial cushions are hypoplastic do point out to a major role for NFATC1 in endocardial cushion formation and proliferation. This role is even highlighted by the inactivation of PPP3CB, which encodes the calcineurin regulatory subunit, specifically in the endocardium and that results in a mirror-image phenotype identical to that of the *Nfatc1*−/− knock-out [28], [29]. This intrinsic requirement for endocardial expression of NFATC1 in endocardial cushion formation is dispensable for endocardial-mesenchymal transformation since in both *Ppp3cb*−/− and *Nfatc1*−/− embryos, mesenchymal cells are found in the cardiac jelly. The Calcineurin/NFAT pathway is however required in myocardial cells to control EMT through the repression of secreted VEGF.

Given the fact that NFATC1 is at the center of valve formation in mammals, we hypothesize that mutations in the gene encoding it would be associated with valve malformations in humans. We have previously shown that a tandem repeat in the intronic region of *NFATC1* is associated with ventricular septal defects but with no valvular phenotype [30]. We therefore screened for such mutations in patients with different valve diseases registered at the congenital heart disease genetics program at the American University of Beirut Medical Center. Results showed 2 novel missense (P66L, I701L) single nucleotide polymorphisms (SNPs) in only one patient with tricuspid atresia. Functional analyses of the mutated protein do show a defect in its cellular localization, transcriptional activities and DNA binding patterns suggesting that the mutations are disease causing.

## Materials and Methods

### Subject Recruitment and DNA extraction

Blood was extracted from registered patients at the Children's Cardiac Registry Center at the American University of Beirut Medical Center (AUB-MC) after signing a consent form approved by the IRB (Protocol Number: Bioch.GN01). Patients included in the study, were evaluated by a pediatric cardiologist. The diagnosis was confirmed at least by echocardiography. Patients with known syndromes (I,e Noonan, DiGeorge, Holt-Oram, Marfan, Alagille, and Char) were excluded from the study. EDTA tubes were used for blood collection and DNA extraction was carried out as previously described [31], [32]. The obtained DNA was quantified at 260 nm and DNA concentration was in the range [400 ng/µl–800 ng/µl].

### Cell Lines

HEK 293T cells (Human Embryonic Kidney cells) and HeLa cells (human cervical cancer cells) were cultured and maintained in Dulbecco's Modified Eagle Media (DMEM) supplemented with 10% Fetal Bovine Serum (PAA) (FBS), 1% Penicillin/Streptomycin and 1% Sodium pyruvate. Incubation was carried out in a humid atmosphere 5% CO2 at 37°C as previously described [31].

### Generation of NFATC1 mutants by PCR-mediated site-directed mutagenesis

After identifying each mutant gene sequence, an oligonucleotide (forward primer) harboring the desired mutation was synthesized in a way to complement the human NFATC1 cDNA (Addgene) subcloned in the pCEP4 expression vector (Invitrogen). The second primer (reverse) was designed in a way that it starts from the same start site of the first primer but extends to the opposite direction. Primers were then phosphorylated and PCR was performed using Site-Directed Mutagenesis kit from FINNZYMES (product code: F-541). The resultant amplicon was ligated and transformed into XL-1 Blue competent bacteria. The generated plasmid was extracted and sequenced to make sure the mutation was incorporated. The primers used for generating the mutants are as follows: 5′ CGGCGCACTCCACCCTGCTGGCCCCGTGC 3′ an its reverse complement for the first mutation , and 5′ CAACGGTAACGCCCTCTTTCTAACCG 3′ and its reverse complement for the second mutation.

### Immunofluorescence

Hela cells were plated in 12-well Costar culture plates on cover slips with 100,000 cells/well. Tranfections were done on the second day of plating using polyethylenimine (PEI) transfecting reagent. Briefly, 2 µg of DNA per well were diluted in 150 µL of NaCl (150 mM, culture grade) in an eppendorf tube, vortexed and then 6 µL of PEI (ratio 1∶3 of DNA to PEI) were added on the mixture of DNA/NaCl, vortexed for 3 seconds and then incubated for 20 minutes at room temperature. The prepared mixture was applied on the cells gently and the medium was replaced on the second day.

Then, immunofluorescence was performed on transfected Hela cells. The cells were first washed for 3 times with PBS 1× (phosphate buffered saline), and then fixed with 4% paraformaldehyde for 20 minutes. Fixed cells were blocked with 3% BSA/PBT (bovine serum albumin/phosphate buffer saline Tween) for 1 hour. The primary antibodies Mouse anti-flag (Flag M2 from Sigma Aldrich) and rabbit anti-HA (Santa Cruz) were used for assessment of subcellular localization of PPP3CA, NFATC1 and its mutants. The primary antibodies were diluted in BSA/PBT and added to the cells with an overnight incubation at 4°C. The cells were then washed in PBT 3 times, and the secondary antibody horse anti-mouse biotinylated or donkey anti-rabbit biotinylated (General Electric) were diluted 1∶500 in BSA/PBT. They were added to the cells for 1 hour at RT with shaking. After washing 3 times with PBT, cells were incubated with Streptavidin Texas red or anti-mouse/anti-rabbit FITC for 1 hour at RT. Staining for the Nuclei was done using the Hoechst dye. The cells were mounted on a rectangular slide containing an anti-fading agent (DABCO), and the slides were examined using an Olympus BH-2 microscope. The nuclear versus cytoplasmic staining was conducted on 3 independent experiments and by assessing a total of 10 fields/per experiment with a total of 125 cells for each mutant and wild type protein.

### Luciferase Assay

HeLa cells were transfected with the 1.4 kbp human DEGS1 or CCND1 promoter coupled to luciferase, the NFATC1 cDNA encoding the different proteins (Wt, P66L, I701L, and P66L/I701L) and/or the constitutively activated PPP3CA and/or GATA5 (Generous gifts from Drs J. Molkentin and M. Nemer) and/or HAND2. Both the DEGS1 and CCND1 promoters were amplified using specific primers and subcloned into the PGL3 Luciferase vector (Invitrogen). The 1.4 Kbp DEGS1 promoter harbors a conserved NFATC1 binding site at −914 bp (5′ TCTTTA**GGAAA**GTCATCTGGTCTGC 3′) in addition to multiple GATA *cis* elements. After 24 hours, cells were washed with PBS (1X) and then lysed with 1X lysis buffer and left on the shaker for 20 minutes at RT. Luciferin (Promega, Cat # E 1501) was prepared according to the manufacturer's protocol. The lysed cells were transferred to a 96 well plate (Costar) to which luciferin was added and the signal was read immediately using the Ascent Fluoroscan in the Molecular Biology Core Facility at AUB.

### Protein over-expression and Western Blots

For over expression experiments, transfections were done using calcium phosphate. Briefly, HEK293T cells were first plated in 100 mm culture plates (Corning) with 70% confluency. On the second day, 20 µg DNA was added to an eppendorf tube; water was added till 200 µl total volume. 400 µl of HBS (Hepes Buffer Sulfate) is added to a tube. Then the mixture of DNA and water is added to the tube. Bubbles are created to promote more DNA precipitation. The mixture is left for 20 minutes at room temperature. The mixture was then applied on cells and after 4 hours the media was replaced. Nuclear protein extracts from HEK293T cells were obtained as previously described. 30 µL aliquots were stored at −80°C . For Western blots, equal amounts of nuclear cell extracts (10 µg protein) were resuspended in 5X laemmli buffers. The samples were boiled for 3 minutes and run on a denaturing SDS-PAGE for 1.5 hours then transferred to a PVDF membrane (Amersham). The membrane was blocked for 45 minutes in 2% non-fat dry milk . After blocking, the membrane was incubated with the primary antibody, Anti- Flag (against NFATC1) or/and anti-HA (against PPP3CA). The antibody was diluted 1∶1000 in 1% non-fat dry milk and the incubation was carried out overnight at 4°C. The membrane was afterwards incubated with the secondary antibody conjugated with horseradish-peroxidase, anti-mouse or anti-rabbit- HRP, diluted 1∶40000. Revelation was done using the Western Lightening Chemiluminescence Kit (Perkin Elmer, Cat # NEL 103). The protein bands were visualized by autoradiography.

### Electrophoretic Mobility Shift Assay (EMSA)

For probe synthesis, two pairs of primers were designed corresponding to the NFAT consensus region 5′ CGCCCAAAGA**GGAAA**ATTTGTTTCATA 3′ (Santa Cruz). The single stranded primers were annealed and labeled with P32 in presence of T4 Kinase. The labeled probe was then run on a non-denaturing 12% Bis-Acrylamide gel (Acrylamide: Bis (38∶2), 1.6%APS, TEMED, water and 1X TBE) at 125 volts for 30 minutes. The gel was exposed to a XOMAT film and the bands corresponding to a double stranded probe were cut accordingly and purified using Costar Spin-X columns (Costar, Cat # 8161) according to the manufacturer's protocol. The probe was then used in gel shift assay experiments.

The nuclear extracts were run on a 6% non-denaturing polyacrylamide gel (Acrylamide: Bis (29∶1), 1.6%APS, TEMED, water and 0.25X TBE) in 0.25X TBE buffer at 200 volts. The reaction consisted of 10 µg of extracts, 4 µl binding buffer (20 mM Tris pH 7.9, 120 mM KCl, 2 mM EDTA, 25 mM MgCl2 and 25% glycerol), 1 µl poly dI/dC (General Electric) and 1 µl of the probe. The reaction was completed to 20 µl with water. After incubation for 20 minutes the samples were loaded and the gel was run for 2.5 hours. The gel was then dried using the BioRad gel dryer (Model 583) for 2 hours at 80°C followed by exposition to a PhosphoImager screen. The results were visualized using the STORM scanner (General Electric). Quantification of the bands was done using TotalLab2010 (General Electric).

### Statistical Analysis

The significance of the luciferase transcriptional assays was analyzed using the one-way Anova single test (p<0.05).

### Bioinformatics

The NFATC1 secondary structures were predicted and visualized by the Discovery Studio program (Acclerys Inc.). Briefly, the human NFATC1 protein sequence was imported from the SWISS database and the secondary amino acid structure was predicted based on the nature and structure of the composing amino acids using already validated approaches by the Discovery Studio. The mutated amino acids were introduced to the same sequence, and the prediction of the structure was carried on using the same approach.

## Results

Patients with various heart valve defects were recruited as part of the ongoing study on the genetics of CHD in the Lebanese population. The subjects' list included a total of 135 patients and 100 control healthy individuals. The distribution of valvular CHDs among patients was as follows: tricuspid atresia (19 patients), pulmonary stenosis (63 patients, 9 of which are strictly valvular), aortic stenosis (5 patiens), pulmonary atresia (9 patients), and mitral atresia (4 patients). In addition, 21 cases of ventricular septal defect and 14 cases of coarctation of the aorta (14 patients) were included (Table 1).

**Table 1:** Number and Phenotypes of the Lebanese Subjects Enrolled in this Study.

TA	19
CoA	14
PA	9
MA	4
AS	5
PS (Valvular)	63 (9)
VSD	21
Control subjects	100

### Heterozygous SNPs in exon 2 and 8 of NFATC1 in one patient with TA

**Figure 1:**
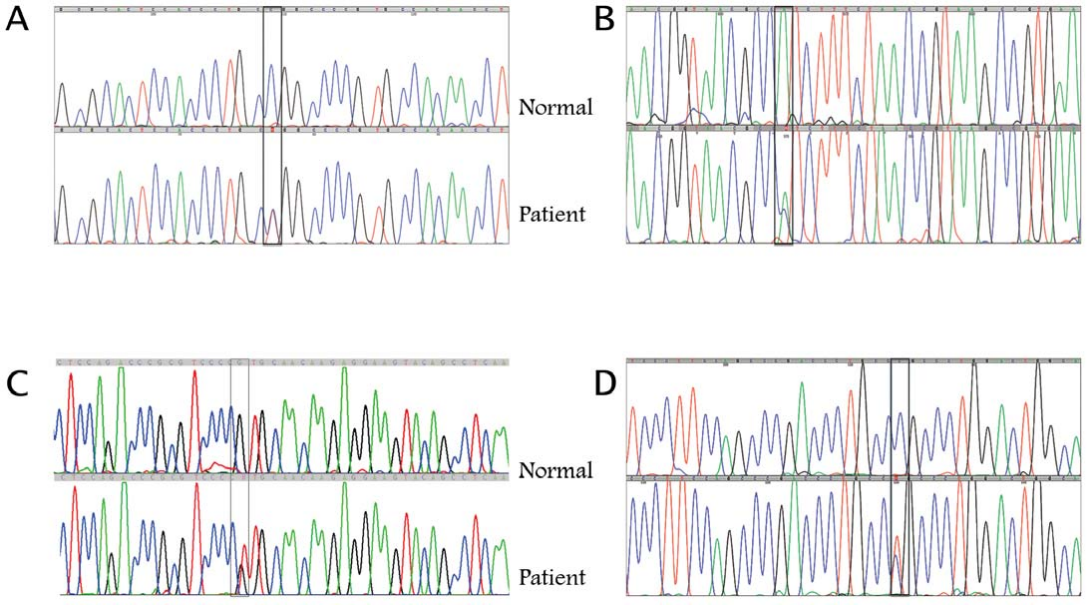
Sequencing results showing the different NFATC1 SNPs. Representative chromatograms of the different missense SNPs in exons 2 and 8 (A and B respectively) and synonymous SNPs in exon 2 and 3 (C and D respectively).The boxed region indicates the place of the polymorphisms in the patient as compared to a normal sequence. In all cases, the SNPs occur on one allele as visualized by overlapping peaks at the indicated position inside the box. In (A) a cytosine is substituted by a thymine, in (B) an adenine is substituted by a cysteine, in (C) a guanine is substituted by a thymine, and in (D) a cytosine is substituted by a thymine.

**Figure 2:**
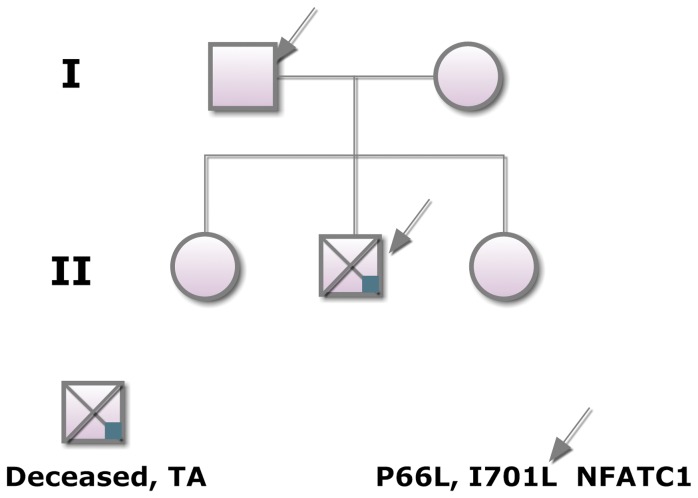
Mendelian inheritance of the different NFATC1 SNPs. Genotype-phenotype correlations showed that in addition to the indexed patient with tricuspid atresia who died at 17 years of age, his “healthy” father carried the four different SNPs. None of the siblings, nor the mother who all are healthy have any of these SNPs.

**Figure 3:**
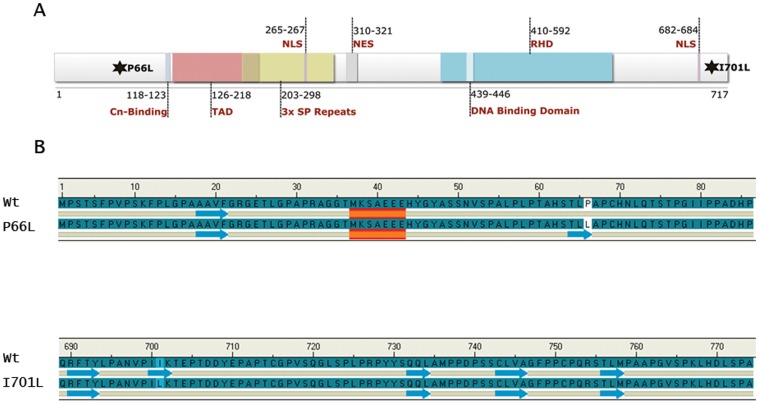
Effects of the two missense SNPs on the structure of the NFATC1 protein. A- The missense SNPs lead to a P66L substitution at the N-terminal region of the protein near the calcineurin-docking site (Cln binding), and to a I701L substitution at the C-terminal region downstream of the Rel Homolgy Domain (RHD). The schematic represents isoform A, the most abundant NFATC1 protein with 717 amino acids, a transactivation domain (TAD) at the N-terminus, and a DNA binding domain at the C-terminus. (NLS = nuclear localization signal, NES = nuclear export signal, and SP = Serine-Proline). B- The NFATC1 secondary structures were predicted and visualized by the Discovery Studio program (Acclerys Inc.). The results demonstrate the formation of a new beta-sheet in the P66L mutant and a deletion of a beta-sheet in the I701L mutant as compared to the wild type protein.

We screened all 8 coding exons of the *NFATC1* gene for the 135 patients. Only one patient (#120) suffering from Tricuspid Atresia was found to have two missense heterozygous SNPs in exons 2 and 8 respectively (Figure 1 A,B). Additional silent SNPs were found in various exons in many patients (data not shown) including patient 120 (Figure 1 C,D). The patient presented to the clinic at the American University of Beirut Medical Center at sixteen years of age with severe hypoxemia probably due to complications of his undiagnosed tricuspid atresia problem over the years. The echocardiography at that stage showed in addition to TA, a D-TGA (transposition of the great arteries), atrial septal defect (ASD), hypoplasia of the aortic arch, and severe pulmonary hypertension. The patient died shortly after hospital admission. Family members of the patient (Figure 2) were screened to check for a Mendelian inheritance of the missense SNPs. Surprisingly the father who is “healthy” with no cardiac phenotype was also found to carry the same SNPs. All other family members including the mother and two siblings were genotypically normal. The T/C nucleotide SNP in exon 2 leads to a Leucine instead of a Proline at position 66 and the A/C nucleotide SNP in exon 8 generates a Leucine instead of Isoleucine at position 701 (Figure 3A). The screening of 100 healthy control individuals didn't show the presence of these SNPs, suggesting they might be disease-causing. Both SNPs were however found in the dbSNP database with the following rsID: rs1481042045 and rs113736099 for the P66L and I701L respectively. The minor allele frequency (MAF) is considerably very low especially for rs1481042045, and *in silico* prediction, using Polyphen-2 shows that this particular SNP is potentially damaging for the protein (Table 2).

**Table 2:** Frequency of the NFATC1 mutations according to the Exome Sequencing Project (ESP).

rsID	Alleles	EA Allele#	AA Allele#	All Allele#	MAF(%)(EA/AA/All)	Amino Acid Position	Polyphen Prediction
rs148104245	T/C	T = 0C = 8598	T = 3C = 4403	T = 3C = 13001	0.0/0.0681/0.0231	LEU,PRO 66/7171	ProbablyDamaging
rs113736099	C/A	C = 10A = 8590	C = 12A = 4392	C = 22A = 12982	0.1163/0.2725/0.1692	LEU,ILE 701/717	Unknown

**rs ID**

dbSNP reference SNP identifier.

**EA Allele Count**

The observed allele counts for the listed alleles in European American population. (delimited by /).

**AA Allele Count**

The observed allele counts for the listed alleles in African American population. (delimited by /).

**Allele Count**

The observed allele counts for the listed alleles in all populations. (delimited by /).

**MAF (%) (EA/AA/All):**

the minor-allele frequency in percent listed in the order of European American (EA), African American(AA) and all populations (All). (delimited by /).

### Disruption of the secondary structure of the P66L and I701L NFATC1 proteins

Bioinformatics secondary structure prediction tools were used to assess the effect of the mutations on the secondary structure of NFATC1 protein. Upon substitution of P with L at position 66, a new beta sheet was formed in comparison with the Wt NFATC1 while a beta sheet was removed at position 701 upon substitution of I with L (Figure 3B). This confirms the *in silico* predictions done by the Polyphen-2 software used by the Exome Variant Server (EVS) database to predict the effect of amino acid substitution on protein function (http://evs.gs.washington.edu/EVS/).

### The NFATC1 double mutant protein is partially retained in the cytoplasm

**Figure 4:**
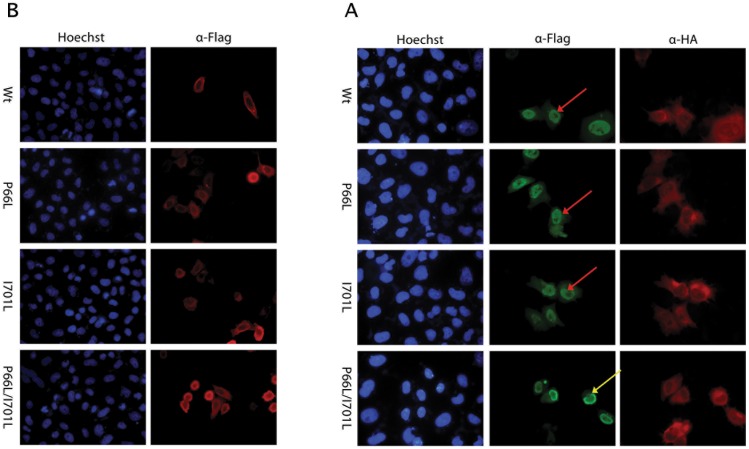
Effect of the NFATC1 missense SNPs on the cellular localization of the protein. A- Immunofluorescence of HeLa cells transfected with plasmids encoding for the Wt NFATC1 and NFATC1 Mutants (P66L, I701L, P66L/I701L). The localization of NFATC1 was visualized using an anti-Flag antibody. Nuclei of cells were visualized using the Hoechst dye (blue color). Wt and NFATC1 mutants localized to the cytoplasm in the absence of PPP3CA (red color). (Magnification ×40). B- Immunofluorescence of HeLa cells transfected with plasmids encoding for the Wt NFATC1 and NFATC1 Mutants (P66L, I701L, P66L/I701L) co-transfected with PP3CA. The localization of NFATC1 was visualized using an anti-Flag antibody (red color) while PP3CA was visualized using anti-HA antibody (green color). Nuclei of cells were visualized using Hoechst dye (blue color). Most of the cells co-transfected with the double NFATC1 mutant were retained in the cytoplasm around the nuclear membrane, whereas in the other cases, the protein was totally translocated to the nucleus. (Magnification ×40). Yellow arrows indicate cytoplasmic (peri-nuclear) staining, while red arrows indicate nuclear staining.

In order to assess the impact of the P66L and I701L mutations on NFATC1 structural and functional properties, site directed mutagenesis was done on a human *NFATC1* cDNA (Isoform A, NP_765978.1) cloned in an expression vector. Three vectors were generated harboring P66L alone (P66L), I701L alone (I701L) and both mutations together (P66L/I701L). The generated plasmids were transfected into HeLa cells to study the cellular localization of the mutated protein. Immunostaining revealed that Wt NFATC1 and NFATC1 mutants are located in the cytoplasm in absence of PPP3CA(Figure 4A). Wt NFATC1, P66l, and I701L translocated to the nucleus when cotransfected with the activated form of PPP3CA (Figure 4B). However, NFATC1 double mutant P66L/I701L failed to translocate to the nucleus in more than 80% of co-transfected cells (Figure 4B).

### Attenuated DNA binding affinity of the mutant NFATC1 mutant proteins

**Figure 5:**
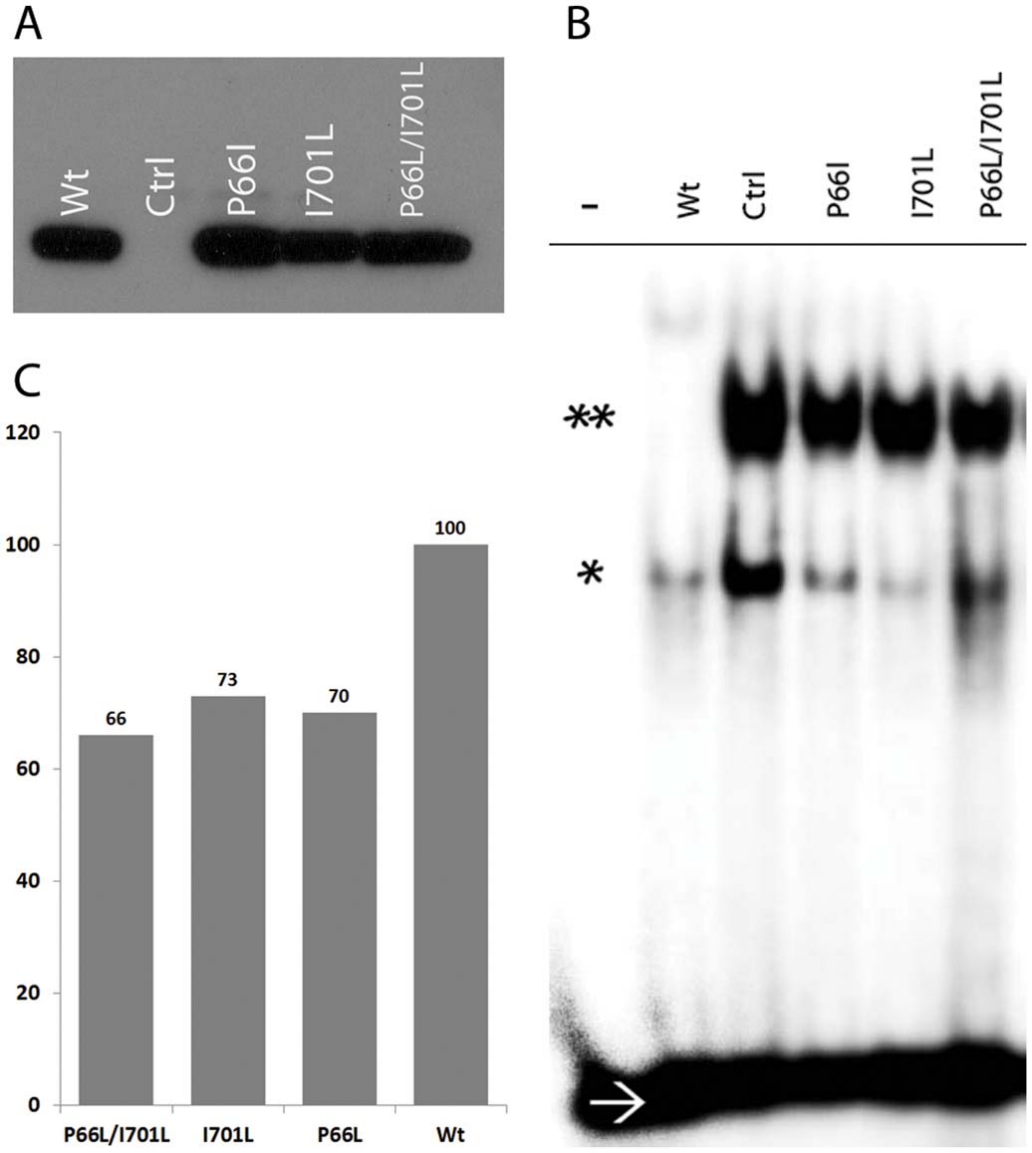
DNA binding affinity of the mutated NFATC1 proteins. A- NFATC1 extracts from HEK 293 cells transfected with Wt NFATC1 and Mutants (P66L – I701L – P66L/I701L) were resolved on an SDS-PAGE prior to gel shift assays. Western blots showed equal amounts of expressed proteins as depicted by the anti-Flag antibody. (Ctrl refers to nuclear extracts from mock-transfected cells). B- EMSA was performed using equal amounts of the overexpressed NFATC1 proteins from HEK 293 cells transfected with Wt NFATC1 and NFATC1 mutants (P66L, I701L, P66L/I701L) and NFAT-consensus binding site as a probe. – ve sign indicates absence of nuclear extracts/* indicates NFATC1 monomer/** indicates NFATC1 Dimer/→ refers to the ^32^P labeled free DNA probe. C- Quantification of the NFATC1 dimers in the EMSA using the TotalLab2010 software from Amersham shows a 30% decrease in DNA binding affinity of the single and double mutant as compared to the wild type NFATC1 protein.

Gel shift assays were carried out to assess the binding affinity of the mutated NFATC1 proteins to an NFAT consensus binding sites. Equal amounts of overexpressed proteins were verified by western blots (Figure 5A), and used for DNA-binding activity. Multiple assays with different amounts of proteins showed a consistent decrease in DNA binding affinity of around 30% for all mutant proteins as compared to the wild type NFATC1 (Figure 5, B,C).

### NFATC1 mutations hampered Calcineurin induced transcriptional activity

**Figure 6:**
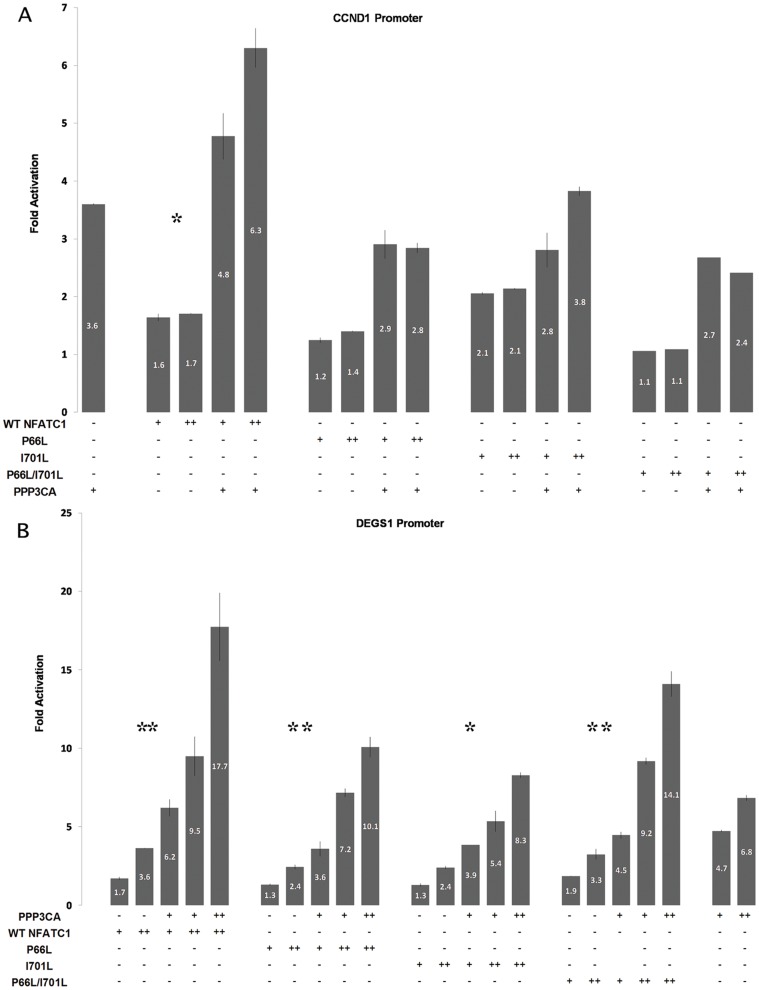
Transcriptional activity of the mutated NFATC1 proteins. A- Wt NFATC1 or NFATC1 Mutants (P66L, I701L, P66L/I701L) were cotransfected with the human CCND1 promoter coupled luciferase reporter construct in the presence or absence of activated clacineurin (PPP3CA) in Hela cells. Six hours post transfection, media was changed and cells were harvested for luciferase assay after 36 hours. Relative luciferase activities are represented as fold activation. The data are the mean of three independent experiments done in duplicates +/− standard deviation. Significance (p<0.05) was assessed using the one-way Anova test. (* p<0.01, ** p<0.05) B- Wt NFATC1 or NFATC1 Mutants (P66L, I701L, P66L/I701L) were cotransfected with the human DEGS1 promoter coupled luciferase reporter construct in the presence or absence of activated clacineurin (PPP3CA) in Hela cells. Six hours post transfection, media was changed and cells were harvested for luciferase assay after 36 hours. Relative luciferase activities are represented as fold activation. The data are the mean of three independent experiments done in duplicates +/− standard deviation. Significance (p<0.05) was assessed using the one-way Anova test. (* p<0.01, ** p<0.05).

In order to assess the impact of the mutations on the regulatory function of NFATC1 protein, transactivation assays using the cyclin D1 (CCND1), and the Degenerative Spermatocyte Homolog 1 (DEGS1) promoter fused to luciferase were performed. HeLa cells were transfected with 1 µg of (DEGS1/luc)/well and increasing concentration of Wt NFATC1 and NFATC1 mutants with or without constitutively activated PPP3CA. The DEGS1 promoter harbors a consensus NFAT binding site at −914 bp in addition to multiple GATA binding sites. The results showed that the Wt NFATC1 is a moderate activator of the DEGS1 promoter with a maximum fold increase of 1.7 (Figure 6A). Upon co-transfection with PPP3CA, the activation of DEGS1 promoter increased to reach 6.2 without attaining a synergistic threshold. This synergy is however observed when the amount of Wt NFATC1 was increased (Figure 6 A). In comparison, the different mutant NFATC1 proteins have a decreased transcriptional activity alone or in combination with PPP3CA.

The same approach was adopted to assess NFATC1 regulation of CCND1 promoter, a recently described *bona fide* target of NFATC1 [33]. The Wt protein showed a dose dependent activation of the promoter that was increased in presence of PPP3CA. NFATC1 mutants (P66l, I701L, and P66L/I701L) showed decreased activation of the promoter that was more significant in the case of the double mutant P66L/I701L (Figure 6 B).

### The NFATC1 double mutant is unable to functionally interact with both GATA5 and HAND2

**Figure 7:**
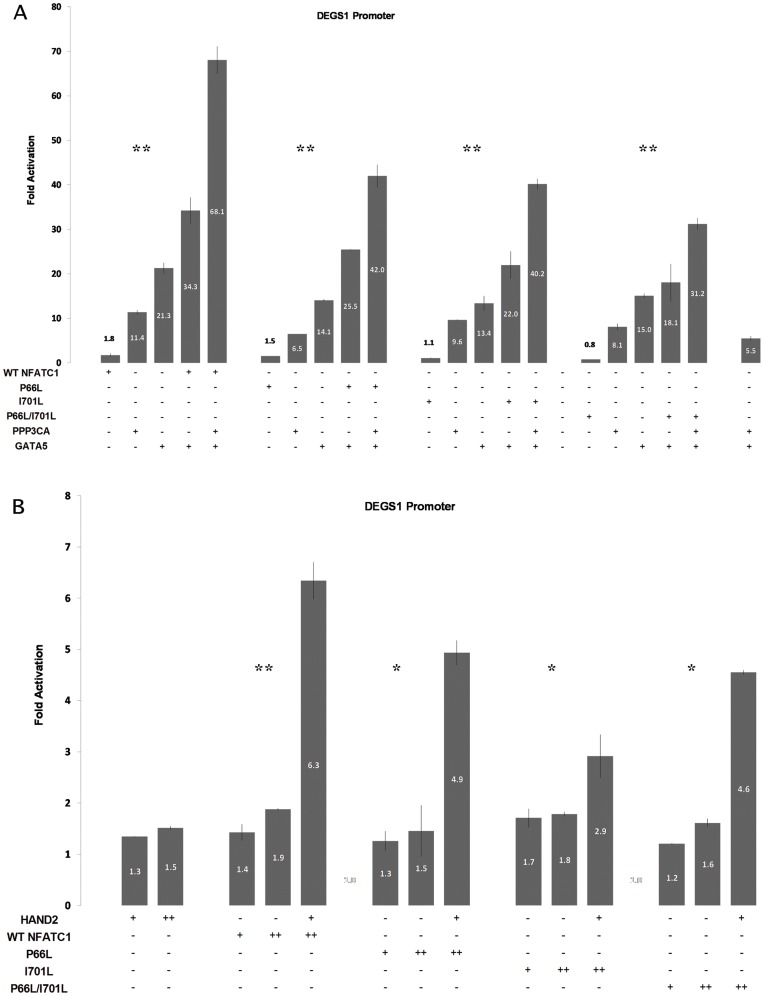
NFATC1 mutations impair functional interactions with GATA5 and HAND2. A- Wt NFATC1 or NFATC1 Mutants (P66L, I701L, P66L/I701L) were transfected with/without HAND2 and the DEGS1 promoter coupled to luciferase reporter construct in Hela cells. Six hours post transfection, media was changed and cells were harvested for luciferase assay after 36 hours. Relative luciferase activities are represented as fold activation. The data are the mean of three independent experiments done in duplicates +/− standard deviation. Wt NFATC1 and HAND2 synergistically activate DEGS1 promoter. This synergy was abrogated in all NFATC1 mutants. Significance (p<0.05) was assessed using the one-way Anova test. (* p<0.01, ** p<0.05) B- Wt NFATC1 or NFATC1 Mutants (P66L, I701L, P66L/I701L) were transfected with/without PPP3CA and with/without GATA5 to assess their combinatorial regulation of the DEGS1 promoter in HeLa cells. Six hours post transfection, media was changed and cells were harvested for luciferase assay after 36 hours. Relative luciferase activities are represented as fold activation. The data are the mean of three independent experiments done in duplicates +/− standard deviation. Wt NFATC1 cotransfected with GATA5 caused a synergistic activation of 35 fold, while transfection of Wt NFATC1 with PPP3CA and GATA5 caused even a stronger synergy reaching 68 fold. The synergestic activation was maintained in all mutants except for P66L/I701L double mutant where the synergy was totally lost. Significance (p<0.05) was assessed using the one-way Anova test. (* p<0.01, ** p<0.05).

Interaction of GATA5 and NFATC1 on DEGS1 promoter was studied based on previous data implicating both proteins in having physical and functional interaction over the endothelin promoter [19]. Hela cells were transfected with GATA5 alone, PPP3CA alone, Wt NFATC1 alone or NFATC1 mutants, a combination of each two, or a combination of the three together. Wt NFATC1 alone, PPP3CA alone, and GATA5 alone resulted in 1.8, 11.4 and 21.5 times fold activation respectively. Wt NFATC1 cotransfected with GATA5 caused a synergistic activation of 35 fold, while transfection of Wt NFATC1 with PPP3CA and GATA5 caused even a stronger synergy reaching 68 fold (Figure 7A). The combination of GATA5 with either the P66L or I701L NFATC1 mutants still yield a synergistic activation of the DEGS1 though at a much reduced magnitude as compared to the Wt. Only the double NFATC1 mutant failed to synergistically interact with GATA5.

On the contrary, the interaction of NFATC1 and HAND2, a recently identified pathway implicated in chronic hypoxia, was totally disrupted over the DEGS1 promoter when any of the NFATC1 mutation was introduced (Figure 7B).

## Discussion

Congenital heart diseases are still the leading cause of death in newborns in addition to being the most frequent congenital diseases in humans [6]. The genetic mechanisms underlying such diseases however, are being unraveled slowly in the last decade because of the tremendous work done on understanding the molecular mechanisms governing cardiac development in numerous organisms [34]. These mechanisms include the collaborative interaction between transcription factors and their occupancy of conserved *cis* regulatory elements on different cardiac-specific promoters. The cloning and functional characterization of the genes encoding these transcription factors have successfully led to the formulation of hypotheses that mutations in these genes could cause heart malformations in humans. More importantly, the available data on genes such as *GATA4*, *NKX2-5* and *TBX5* do point to a dose-dependent genotype-phenotype correlation whereby haploinsufficiency is by itself diseases-causing [31], [35], [36], [37], [38]. Our results go along with what is published in that regard by adding the *NFATC1* gene to the list of mutated genes linked to congenital heart disease in humans, particularly valve diseases.

### NFATC1 haploinsufficiency and Tricupid Atresia

We have shown two heterozygous mutations on one allele of the NFATC1 gene in one patient with tricuspid atresia out of 19. The fact that the double mutation is also found in the father who has a normal phenotype argues for incomplete penetrance, a phenomenon seen in other genes encoding transcription factors involved in cardiac and non-cardiac congenital diseases. One such example is the Arg25Cys mutation, which was shown to abrogate the transcriptional activity of the NKX2-5 protein and yet has reduced penetrance depending on the population study groups [39], [40]. In mice, the Holt-Oram syndrome recreated with the heterozygous Tbx5 model is the best example of a dosage dependent phenotype-genotype correlation. In fact, null mice for both Tbx5 alleles showed a very severe cardiac phenotype leading to early embryonic lethality, while mice carrying only one Tbx5 allele display a spectrum of phenotypes recapitulating the ones observed in humans [41]. Unfortunately, in our case the indexed-patient was evaluated for the first time at the age of 16 years at our center when he presented with severe cyanosis and complications of his condition which was not well taken care of at earlier stages and had led to the his death few days after his admission to the hospital. Exon by exon sequencing of different genes encoding transcription factors, including *GATA4*,*5*,*6*, *TBX5*,*20*, *NKX2-5*, *PITX2*, and *NFATC1* was carried out on the whole family and none except *NFATC1* showed polymorphisms that could be disease causing. We cannot exclude however, that other not tested gene(s) could also be mutated and carried on the maternally inherited allele, and that the combination of such mutations is responsible for the observed phenotype. Alternatively, we could postulate that the father who is phenotypically normal carries the two mutations on separate alleles and that during spermatogenesis a cross-over did occur leaving the two mutations on one allele inherited by the patient, and another normal inherited by the other children. Structurally, the two mutations leading to amino acids substitution at both the N and C terminal of the protein were predicted to be pathogenic, and in our *in vitro* analysis we did show that the double mutation affects both the transcriptional activity and the localization of the protein. At the subcellular localization level, most of the NFATC1 double mutant proteins failed to translocate to the nucleus when co-expressed with constitutively active calcineurin. Although the mutation is not within the calcineurin docking site, we do suggest that the distorted structure of the protein doesn't allow proper dephosphorylation of its N-terminal domain [16], [42], [43]. This is supported by the results obtained in gel shift experiments whereby the DNA binding activity was significantly reduced by 30–40% although the same amount of overepressed proteins was used for both wild type and mutant NFATC1. On the other hand, the structure function analysis done on the most expressed isoform, Isoform A, does also mask a possible effect the mutation I701L could have on the sumolation process on isoform C which occurs on K702 [44].

### The NFATC1 P66L/I701L double mutant: an orphan partner?

**Figure 8:**
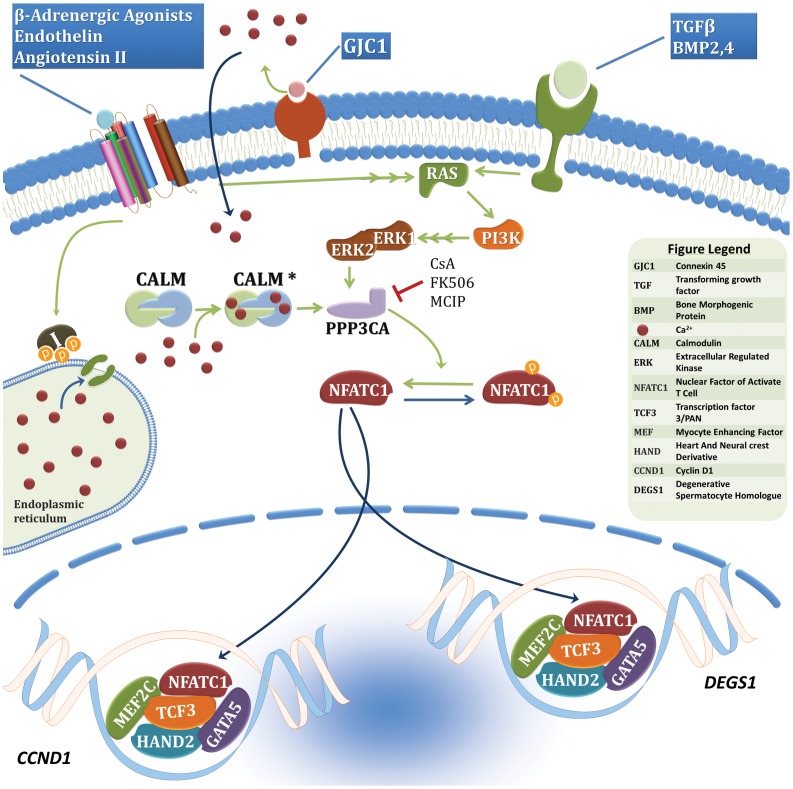
Hypothetical pathway involving NFATC1 in endocardial cushion proliferation and valve maturation.

NFATC1 is a weak transcription factor although it has a specific and strong DNA affinity. Its activity is however enhanced by its interaction with ubiquitously and/or tissue-specific transcription factors like members of the AP-1 and GATA families [21], [45]. GATA5 was previously shown to be a strong partner of NFATC1 and its recent inactivation in mice did show that the embryos develop aortic stenosis one of the most frequent valve abnormalities [19], [46]. The observed phenotype in mice involves the formation of a bicuspid aortic valve instead of a tricuspid one suggesting a role of GATA5 similar to that of NFATC1 in the proliferation of valve precursors and final remodeling part. Our results go in parallel with this suggested role, since the interaction of GATA5 and NFATC1 is relatively hampered by the double mutation. In fact, the functional synergy between both proteins was reduced by 50% over the DEGS1 promoter, which was recently shown by our group to be directly regulated by NFAT and HAND2 in chronic hypoxia, a mouse model mimicking cyanotic CHD including Tricuspid Atresia (unpublished data). The HAND2/NFATC1 interaction is also severely affected by the double mutation suggesting a combinatorial interaction between GATA5/NFATC1/HAND2 in a common pathway regulating endocardial cushion formation and valve maturation. One could argue however, that the fact the double mutant is trapped in the cytoplasm might cause the observed inhibition. Nevertheless even with higher doses of transfected mutant vectors, the observed synergy with the wild type protein couldn't be recapitulated. Our hypothetical model would involve regulation of downstream target genes like cyclin D1, which was previously shown to be a direct target for GATA and NFATC1 proteins in the early phases of endocardial cushion proliferation (Figure 8). In fact, in human pulmonary valve endothelial cells, NFATC1 activates in vitro endothelial-specific genes ultimately leading to their proliferation [47]. Furthermore, NFATC1 promotes cell cycle progression in 3T3-L1 cells showing altered expression of cell cycle genes including high levels of cyclin D1 [48]. On the other hand, DEGS1 would be ideal factor involved in valve maturation whereby apoptosis is a key event. In fact, DEGS1 is known to be involved in *de novo* ceramide production, an obligate path leading to apoptosis.

This hypothetical pathway needs to be supported however by an *in vivo* knock-in model for NFATC1 and a cardiac/endocardial conditional knock-out for DEGS1.
